# Intestinal microbial diversity in female rhesus (*Macaca mulatta*) at different physiological periods

**DOI:** 10.3389/fmicb.2022.959315

**Published:** 2022-09-26

**Authors:** Yanyan Li, Fengmei Yang, Lixiong Chen, Suqin Duan, Weihua Jin, Quan Liu, Hongjie Xu, Wei Zhang, Yongjie Li, Junbin Wang, Zhanlong He, Yuan Zhao

**Affiliations:** Institute of Medical Biology, Chinese Academy of Medical Sciences and Peking Union Medical College, Kunming, China

**Keywords:** rhesus monkeys, intestinal, microbial diversity, diversity, menstrual cycle

## Abstract

To explore the relationship between the changes in the physiological period and the fecal microbial population of female rhesus monkeys by measuring microbial composition of fecal samples and the serum hormones. Blood and fecal samples were collected from six female adult rhesus monkeys during the menstrual period (MP), ovulation period (OP), and Luteal period (LP). Serum estradiol (E2) and progesterone (P) levels were determined by the chemiluminescence method and the stool samples were subjected to high-throughput 16S rRNA sequencing. The highest level of E2 and P secretions were during the MP, and LP, respectively. Stool samples produced valid sequences and the number of operational taxonomic unit/OTU was: 810056/3756 (MP), 845242/4159 (OP), 881560/3970 (LP). At the phylum level, the three groups of Firmicutes and Bacteroides accounted for > 95%. The dominant flora at the LP was Bacteroides (53.85%), the dominant flora at the MP and OP was Firmicutes, 64.08 and 56.53%, respectively. At the genus level, the dominant genus at the LP was *Prevotella*, the dominant genera at the MP were *Prevotella, Oncococcus, Streptococcus*, and *Kurtella*. The dominant genera at OP were *Prevotella* and *Nocococcus*. At the phylum level, P levels were negatively correlated to Firmicutes, Actinomycetes Actinobacteria, and Fibrobacteres, but positively correlated to Bacteroidetes. Likewise, E2 was positively correlated to Proteobacteria but negatively correlated to Euryarchaeota. At the genus level, P hormone showed a significant correlation with 16 bacterial species, and E2 was significantly correlated to seven bacterial species. Function prediction analysis revealed a high similarity between the MP and OP with six differentially functional genes (DFGs) between them and 11 DFGs between OP and LP (*P* < 0.05). Fecal microbiota types of female rhesus monkeys varied with different stages of the menstrual cycle, possibly related to changes in hormone levels.

## Introduction

The intestinal flora plays a very important role in the host’s physiological homeostasis, which can be affected by factors such as genetics, environment, health, age, gender, race, diet, and lifestyle (Snigdha et al., 2021; Wang et al., 2021). Intestinal microbes and their metabolic products can affect other organs and systems leading to diseases such as inflammatory bowel diseases, obesity, diabetes, liver diseases, chronic heart diseases, cancers, HIV, and autism (Zhang et al., 2015; Matson et al., 2021; Yang et al., 2021). Apart from external environmental factors (antibiotics, diet, stress, injury, illness, etc.), the genetic and epigenetic factors can also affect the diversity and function of the intestinal flora that may disorder the symbiosis and result in diseases such as neurodegenerative diseases [Alzheimer’s disease (AD), Parkinson’s disease and multiple sclerosis] (Choi et al., 2018), metabolic diseases (osteoporosis, obesity, and autoimmune disease such as rheumatoid arthritis) (Ridlon et al., 2013a), diabetes (Knip and Siljander, 2016), digestive system diseases (liver disease, inflammatory bowel disease, functional gastrointestinal disease, gastrointestinal tumors, etc.) (Ratner, 2015; Wahlström et al., 2016).

The secretion of the pituitary gland regulates the cyclical changes of the endometrium forming the menstrual cycle. Reproductive hormone levels vary with the periodic changes in the menstrual cycle. During a normal and healthy pregnancy, the body undergoes a large hormonal, immune, and metabolic change. Similarly, from early to late pregnancy, the intestinal flora also undergoes tremendous changes (Koren et al., 2012). Studies comparing the individual intestinal flora in mice showed that metabolic changes such as hyperglycemia, insulin resistance, and weight gain are related to changes in the composition of intestinal flora during pregnancy (Curtis et al., 2012). Likewise, studies reported that the bacterial community of captive rhesus monkeys becomes more diversified with significantly different bacterial compositions during lactation and menstrual periods (Hallmaier-Wacker et al., 2019). At present, there are rare reports about the changes in intestinal microbes during the menstrual cycle. Accordingly, to explore the inter-relationship between the menstrual cycle and the microbial populations, this study determined the serum estradiol (E2) and progesterone (P) levels to analyze the composition and function of microorganisms in the stool samples of rhesus monkeys during the menstrual cycle.

## Materials and methods

### Experimental animals

Six healthy rhesus monkeys (female, 6–12 years old) were obtained from the Institute of Medical Biology, Chinese Academy of Medical Sciences School Primate Research Center [production license number: SCXK (Dian) 2020-0005; use license number: SYXK (Dian) 2020-0008]. The viral and intestinal pathogens specified by the national standard were eliminated before the experiment. The six monkeys reared separately in single cages, fed with the same feed every day with *ad libitum* drinking water. The rhesus monkey cage was cleaned daily and disinfected regularly. Three menstrual cycles of the experimental rhesus monkeys were recorded to determine the menstrual period (MP), ovulation period (OP), and luteal phase (LP). The experimental content was reviewed and approved by the Institutional Animal Care and Use Committee of the Institute of Medical Biology, Chinese Academy of Medical Sciences (ethics number: DWSP201810002). The experiments were performed following the 3R principle of experimental animal Humane care.

### Determination of serum hormones

The blood samples from the experimental rhesus monkeys, one sample/animals for a total of 18 samples were collected during the MP, OP, and LP (Supplementary Table 1). After the hind limbs of experimental monkeys were shaved and sterilized with alcohol, the blood of the small saphenous vein was collected in a blood collection tube filled with separating gel and coagulant (Shandong Aosaite Medical Instrument Co., Ltd., Chengwu County), were centrifuged at 3,000 rpm for 10 min at 4°C. The separated serum was stored at –80°C in small batches to avoid repeated freezing and thawing. Serum levels of E2 and P hormones were determined using the chemiluminescence method (Li et al., 2019). Before samples were tested and analysed, perform calibration, quality control, maintenance, etc. on the instrument according to the requirements of the operating instructions, and perform performance verification on hormones with reference to CLSI standard documents and guidelines. Precision and accuracy testing, analytical range testing were tested to ensuring that the instrumental errors were within acceptable limits, then serum samples were tested.

### DNA extraction and sequencing

One fecal sample/animals for a total of 18 samples were collected during the MP, OP, and LP. Fresh feces were collected from the middle aseptically within 10 min of excretion and quickly transported to the laboratory –80°C freezer in an icebox. Microbial DNA was extracted using the HiPure Stool DNA Kits (D3141, Magen, Guangzhou, China) from the stool sample and the V3-V4 region of 16S rDNA was amplified with barcode-specific primers; 341F: 5’-CCTACGGGNGGCWGCAG-3’, and 806R: 5’-GGACTACHVGGGTATCTAAT-3’. The PCR reaction was carried out in a 50 μL reaction volume with TransGen High-Fidelity PCR SuperMix (TransGen Biotech, Beijing, China), 0.2 μM forward and reverse primers, and 5 ng template DNA. Amplicons were evaluated with 2% agarose gels and purified using the AxyPrep DNA Gel Extraction Kit (Axygen Biosciences, Union City, CA, USA) according to the manufacturer’s instructions. Sequencing libraries were generated using SMRTbell TM Template Prep Kit (PacBio, Menlo Park, CA, USA) following manufacturer’s recommendation. The library quality was assessed with Qubit 3.0 Fluorometer (ThermoFischer Scientific, USA) and FEMTO Pulse system (Agilent Technologies, Santa Clara, CA, USA). The libraries were sequenced on the Hiseq2500 PE250 platform. The raw reads were deposited into the NCBI Sequence Read Archive (SRA) database (BioProject: PRJNA869047).

### Alpha diversity analysis and beta diversity analysis

To ensure the statistical reliability and biological validity of the data, the original data was cleaned for low-quality reads, and re-filtered after assembly to obtain the valid sequences. Sequences with > 97% identity were clustered into OTUs using the Uparse software (Edgar, 2013). A dilution curve was constructed based on the number of OTUs and alpha diversity analysis was performed to calculate the Chao1, Ace, Coverage, Shannon, and Simpson indices were calculated in QIIME (Caporaso et al., 2010) to determine the sample microbial diversity. Alpha index comparison between groups was calculated by Welch’s *t*-test in R project Vegan package (Oksanen et al., 2010). PCA (principal component analysis) was performed in R project Vegan package (Oksanen et al., 2010). Between groups Venn analysis was performed in R project VennDiagram package (Chen and Boutros, 2011) (version1.6.16). Next, species annotations were performed, differences between groups were compared at different taxonomic levels.

### Function prediction

The KEGG pathway analysis of the OTUs was inferred using PICRUSt (Douglas et al., 2019). Microbiome phenotypes of bacteria were classified using BugBase. FAPROTAX database (Functional Annotation of Prokaryotic Taxa) and associated software were used for generating the ecological functional profiles of bacteria. Analysis of function difference between groups was calculated by Welch’s *t*-test in R project Vegan package.

### Environmental factor analysis

Redundancy analysis (RDA) was executed in R project Vegan package (Oksanen et al., 2010) to clarify the influence of environmental factors on community composition. Pearson correlation coefficient between environmental factors and species was calculated in R project psych package (version 1.8.4) (Revelle and Revelle, 2015). Heatmap and network of correlation coefficient were generated using Omicsmart.

### Statistical methods

Data are expressed as mean ± standard deviation. The SPSS 23.0 statistical software (IBM) was used to analyze the E2 and P hormone levels, community structure, and differences in species composition. *P* < 0.05 indicates that the difference is statistically significant.

## Results

### Serum estradiol and progesterone hormone levels in the breeding season

Serum levels of E2 and P hormones were measured during the MP, OP, and LP. We found that the level of E2 secretion was highest during MP and lowest during LP with a significant difference between the two phases (*P* < 0.05). The level of P secretion was highest in LP and lowest in MP and showed significant differences between the OP, LP, and MP (*P* < 0.05; Supplementary Table 2).

### Sequencing data analysis

**FIGURE 1:**
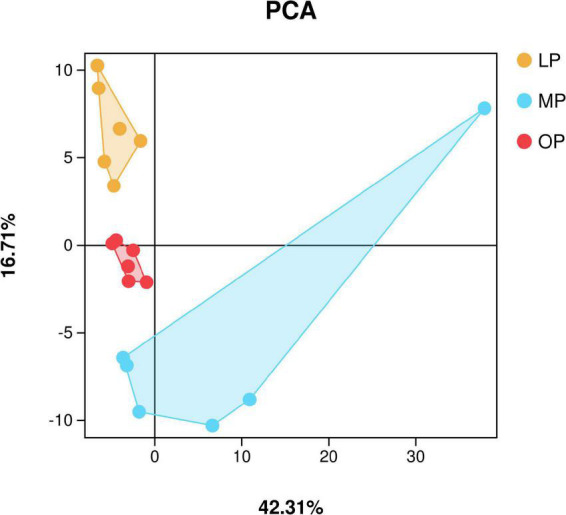
Principal component analysis (PCA).

In total, 2,536,858 sequences were obtained from fecal samples at MP, OP, and LP, which were clustered based on > 97% identity criteria. Next, alpha diversity analysis was used to calculate the Chao1, Ace, Coverage, Shannon, and Simpson indices to determine the richness and diversity of the sample flora. The results show that the sequencing coverage in each group was > 99.9% (Table 1). Also, Shannon’s exponential dilution curve showed a plateau in all groups suggesting that the sequencing volume became saturated and covered most of the species in the sample flora (Supplementary Figure 1). The PCA analysis results are shown in Figure 1. The Shannon index of the OP group was significantly higher than that of the MP and LP groups (*P* < 0.05), and the Simpson index of the OP group was significantly higher than that of the LP group (*P* < 0.05).

**TABLE 1:** Comparison of fecal microbial alpha diversity index at different stages of the menstrual cycle in rhesus monkeys.

Group	OTU	Chao1	Ace	Simpson	Shannon	Coverage
MP	626.00 ± 54.77	728.60 ± 79.63	715.96 ± 66.13	0.93 ± 0.05^ab^	5.69 ± 0.76^b^	0.9991 ± 0.0002
OP	693.17 ± 75.48	778.79 ± 63.69	768.85 ± 65.02	0.97 ± 0.01^b^	6.59 ± 0.30^a^	0.9992 ± 0.0000
LP	661.67 ± 47.42	761.49 ± 68.27	739.60 ± 61.26	0.96 ± 0.01^a^	6.10 ± 0.18^b^	0.9992 ± 0.0002

Three groups of data comparison, for the same detection index, marked with the same letter means no significant difference (*P* > 0.05), different letters means significant difference (*P* < 0.05). Shannon index in OP group was significantly higher than that in MP and LP groups (*P* < 0.05), but LP and OP had no significant difference. The Simpson index of OP group was significantly higher than that of LP group (*P* < 0.05), there was no significant difference between MP and OP, MP and LP.

### Change in fecal microbial composition during the menstrual cycle

**FIGURE 2:**
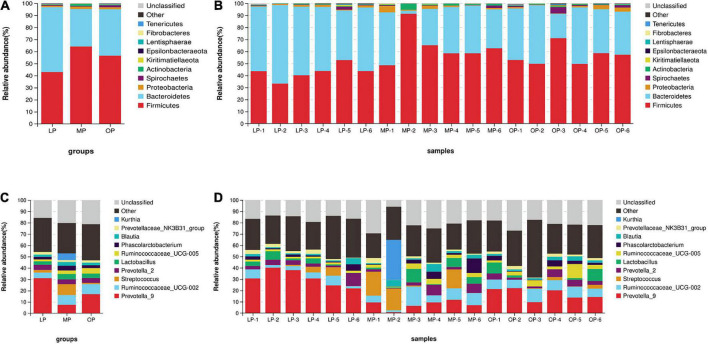
The distribution of fecal microorganisms at the phylum levels at the respective stages of the menstrual cycle. The average of six samples **(A)** and each of the individual six samples at the phylum level **(B)**. The distribution of fecal microorganisms at the genus levels at the respective stages of the menstrual cycle. The average of six samples **(C)** and each of the individual six samples at the genus level **(D)**.

Comparison of flora composition at the phylum level (an average of all six monkeys) revealed that the Bacteroidetes (53.85 ± 7.74%), Firmicutes (42.90 ± 6.32%), and Proteobacteria (1.15 ± 0.54%), in total 97.90 ± 1.67%, accounted for the largest proportion in the LP group. Likewise, the dominant flora of the MP group were Firmicutes (64.08 ± 8.89%), Bacteroides (31.18 ± 14.92%), Proteobacteria (1.89 ± 1.68%), and Actinobacteria (1.66 ± 1.59%), in total 98.81 ± 0.84%. In the OP group, Firmicutes (56.53 ± 8.05%), Bacteroides (38.51 ± 10.41%), Spirochetes (1.64 ± 1.58%), and Proteobacteria (1.62 ± 1.42%), in total 98.29 ± 1.11%, were the most dominant flora (Figures 2A,B). Comparisons at the genus level identified a total of 10 genera. The genera (an average of all six monkeys) with relative abundance > 5% included *Prevotella_9, Ruminococcaceae_UCG-002, Streptococcu*s, and *Kurthia*. The dominant genus of the LP group was *Prevotella* (30.96 ± 7.21%); the dominant genera of the MP group were *Prevotella* (7.49 ± 3.80%), *Ruminococcaceae_UCG-002* (8.57 ± 5.24%), *Streptococcus* (9.71 ± 9.17%), and *Kurthia* (5.91 ± 5.65%); the dominant genera of the OP group were *Prevotella* (16.86 ± 5.01%), and *Ruminococcaceae*_UCG-002 (8.91 ± 1.66%) (Figures 2C,D).

### Comparison of fecal microbes at menstrual period, ovulation period, and luteal period

Venn diagram revealing the shared microorganisms in fecal samples of respective stages (among LP and MP, LP and OP, MP and OP, and three groups) is shown the number of OTUs were 635, 710, 636, and 604, respectively (Supplementary Figure 2).

**FIGURE 3:**
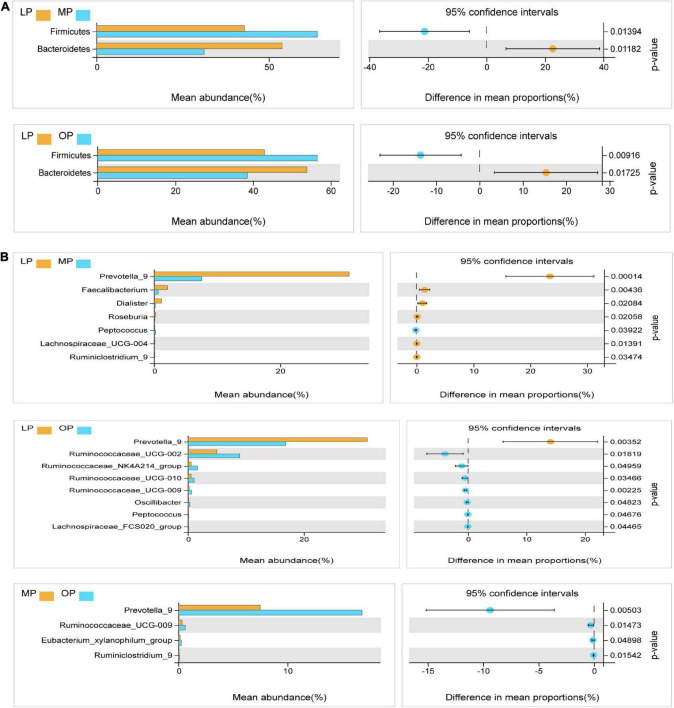
Analysis of the microflora differences at the phylum levels at the respective stages of the menstrual cycle **(A)**. Analysis of the microflora differences at the genus levels at the respective stages of the menstrual cycle **(B)**.

Next, Welch’s *t*-test was used for the differential analysis of the fecal flora at the respective stage. The results showed that at the phylum level, the proportion of Firmicutes was significantly higher in the MP and OP groups than in the LP group (*P* < 0.05); the proportion of Bacteroides was significantly higher in the LP group than in the MP and OP groups (*P* < 0.05) (Figure 3A). At the genus level, a comparison of LP and MP revealed that the average abundance of *Prevotella, Faecalibacterium, Dialister, Roseburia, Lachnospiraceae_UCG-004, Ruminiclostridium* genus was significantly higher in the LP group than in the MP group (*P* < 0.05). Likewise, the proportion of *Peptococcus* in the MP group was significantly higher than in the LP group (*P* < 0.05). Comparison of LP and OP data showed that the proportion of *Prevotella_9* was significantly higher in the LP group than in the OP group (*P* < 0.05). *Ruminococcaceae_UCG-002, Ruminococcaceae_NK4A214_group, Ruminococcaceae_UCG-010, Ruminococcaceae_UCG-009, Oscillibacter, Peptococcus*, and *Lachnospiraceae_FCS020_group* were significantly higher in the OP group than in the LP group (*P* < 0.05). Comparison of OP and MP revealed that *Prevotella_9, Ruminococcaceae_UCG-009, Eubacterium_xylanophilum_group*, and *Ruminiclostridium_9* were significantly higher in the OP group than in the MP group (*P* < 0.05) (Figure 3B).

### Impact of environmental factors

**FIGURE 4:**
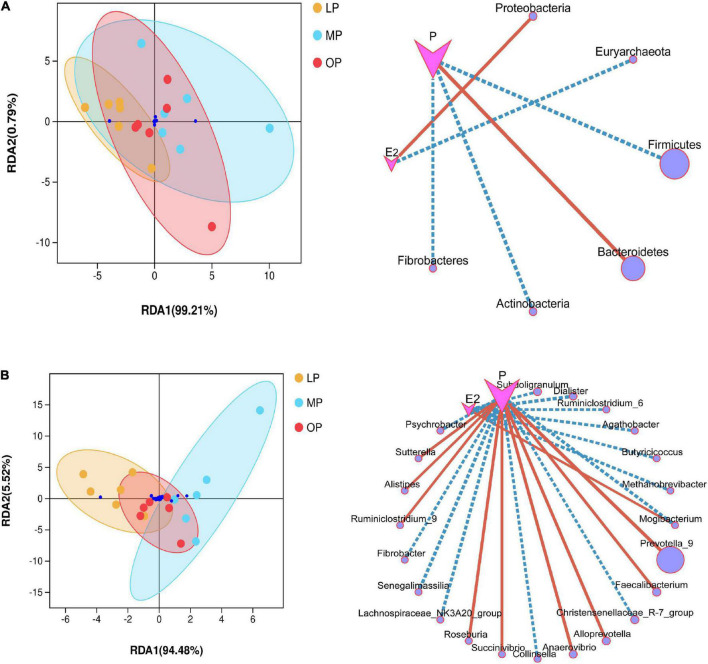
Environmental factor analysis. Redundancy analysis (RDA) and correlation network diagram at the phylum **(A)** and genus **(B)** levels.

Environmental factor analysis revealed the relationship between hormone levels, samples, and microbial communities. RDA analysis is shown in Figure 4. At the phylum level, progesterone is negatively correlated with Firmicutes, Actinobacteria, Fibrobacteres, but positively correlated with Bacteroidetes; Estradiol is positively correlated with Proteobacteria, but negatively correlated with Euryarchaeota (not shown in the abundance diagram) (Figure 4A). At the genus level, estradiol showed a significant correlation with seven bacterial species, among which *Mogibacterium* was significantly positively correlated, while the other six species were negatively correlated. Progesterone showed a significant correlation with 16 bacterial species, including a significant positive correlation with nine bacterial species such as *Prevotella_9*, and a significant negative correlation with seven bacterial species (Figure 4B). The environmental contribution analysis showed that, at the phylum level, the environmental contribution of progesterone was high (28.17%), while, at the genus level, E2 and progesterone contributed 5.23 and 15.15%, respectively (Supplementary Figure 3). This indicates that progesterone contributed more to the total variation of species distribution.

### Functional analysis

Based on the gene type and abundance information of the known microorganisms, KEGG pathway enrichment analysis was performed to predict the key pathways at different physiological periods of the menstrual cycle.

**FIGURE 5:**
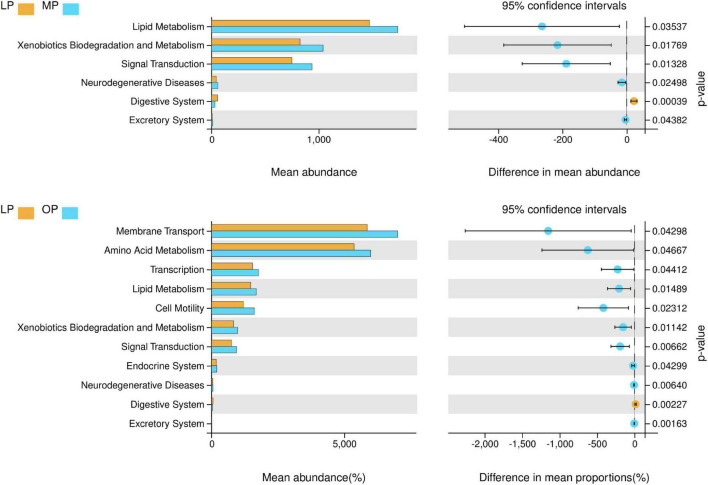
Analysis of differential function at different stages of the menstrual cycle (level 2) (*P* < 0.05).

The results showed that the LP, MP, and OP groups of microbes are rich in functions such as metabolism, signal transduction, membrane transport, transcription, and cell motility. Comparing the three groups, the LP group was rich in glycan biosynthesis and metabolism functions; The MP group had rich functions such as membrane transport, carbohydrate metabolism, amino acid metabolism, lipid metabolism, xenobiotics biodegradation and metabolism, and metabolism of other amino acids. The MP group had rich functions such as replication and repair, translation, energy metabolism, metabolism of cofactors and vitamins, nucleotide metabolism, folding, sorting and degradation, cell motility, and cell growth and death (Supplementary Figure 4). The functional differences of fecal microorganisms in different physiological periods indicate that (Figure 5), the functions such as lipid metabolism, xenobiotics biodegradation and metabolism, signal transduction, neurodegenerative diseases, and excretory system are significantly higher in MP than LP, while digestive system is significantly lower (*P* < 0.05). Likewise, the functions such as membrane transport, amino acid metabolism, transcription, lipid metabolism, cell motility, xenobiotics biodegradation and metabolism, signal transduction, endocrine system, neurodegenerative diseases, and excretory system are significantly higher during OP than in LP, while the digestive system is significantly lower (*P* < 0.05).

## Discussion

The current research on the factors affecting the composition of the gut microbial population of non-human primates mainly focuses on the host species phylogeny, diet, age and sex, social interaction, differences in intestinal segments, health status, etc. (Ochman et al., 2010; Degnan et al., 2012; Moeller et al., 2013; Sun et al., 2016). In addition, the host-related factors (such as genetics, physiology, and reproduction status) and external factors (such as temperature, altitude, and habitat fragmentation), vertical transmission between parents, population density, and mutual contact between species also affect the composition of host intestinal microbes (Ley et al., 2005; Koren et al., 2012). Most studies on sex steroid hormones and microbial changes have focused on vaginal microflora, but produced inconsistent data. Some studies showed that the vaginal microbial population changes during the female reproductive cycle (Uchihashi et al., 2015; Miller et al., 2017), while other studies did not find so (Chaban et al., 2014; Lorenzen et al., 2015). Another report found that the gut microbiota of female mice did not change during the entire estrus cycle (Wallac et al., 2018). However, so far, there is no report about the changes in the fecal microbial populations during the menstrual cycle of non-human primates. Several studies showed that hormones and host microorganisms can influence each other (Yurkovetskiy et al., 2013). Sex steroid hormones can affect the structure, diversity, activity, growth, reproduction, intestinal flora, thereby regulating the host metabolic activities and health. For example, Elin et al. (2016) explored the effects of hormones on the intestinal microbial structure of 89 common hybrid mice by removing the gonads and found that sex steroid hormones caused significant differences in the intestinal flora. Meanwhile, microorganisms also can affect the host endocrine system (Hughes and Sperandio, 2008). The reproductive hormone receptors in microorganisms such as P4 and E2 can regulate the growth and metabolic activity of intestinal microorganisms (Campbell-Thompson et al., 2001; Konstantinopoulos et al., 2003). In addition, intestinal microbes in mice, humans, fish, and other animals (Cima et al., 2004; Wang and Ge, 2004; Sidler et al., 2011; Bouguen et al., 2015) produce the sex steroid hormone synthase and can metabolize the sex steroid hormones (Ridlon et al., 2013b). The normal human intestinal microbiota is mainly composed of Firmicutes, Proteobacteria, Bacteroidetes, Actinobacteria, of which > 90% are Firmicutes and Bacteroidetes (Pan et al., 2021). This study analyzed the types of microorganisms in the stool samples of rhesus monkeys during MP, OP, and LP and found that at the phylum level, the dominant bacterial groups in all the three stages were Firmicutes and Bacteroides, accounting for > 95%. Studies showed that if the stool contains a certain amount of progesterone, the relative abundance of Firmicutes decreases, while Proteobacteria increases (Xue et al., 2017). *In vitro* studies confirmed that progesterone reduces the diversity of microbial composition, but promotes the growth of Bacteroides and Bifidobacteria (Nuriel-Ohayon et al., 2019). In this study, the level of P in the LP group was higher compared with the MP and OP groups, which explains the significantly lower proportion of Firmicutes in the LP group. At the genus level, there was little difference in the composition of the flora during the MP and OP, but both showed quite different flora in the LP. This may be related to the high level of P secretion during the LP and the P induced bacterial proliferation (Xue, 2017). This study found that progesterone promotes the proliferation and growth of 9 bacterial genera including Bacteroidetes and *Prevotella_9*.

The functional prediction analysis showed that the digestive system function was significantly higher in the LP than in the MP and OP, which may be related to Prevotella and Succinivibrio. Prevotella is often closely related to the host’s food digestion. These bacteria can help the leaf-eating slow loris monkeys to degrade the structural carbohydrates in the leaves (Amato et al., 2014, 2015). Likewise, succinivibrio species facilitate the decomposition of plant cellulose into acetic and succinic acids (Sun et al., 2016). Notably, several reports showed the increased incidence of gastrointestinal diseases in healthy women during the MP and OP (Kennedy et al., 2011). P4 and E2 can inhibit the activity and growth of Helicobacter pylori and kill it through bacteriolysis playing a protective role in Helicobacter pylori-induced chronic gastroenteritis, gastric cancer, and other diseases (Hosoda et al., 2011). Interestingly, this study found that P levels are significantly positively correlated with Prevotella and Succinivibrio; Prevotella is the dominant genus in the LP group (30.96%), while the MP and OP groups had only 7.50 and 16.86% of Prevotella, respectively. Therefore, the function of the digestive system in the LP is significantly higher than in other periods. The Firmicutes/Bacteroidetes ratio is an energy output indicator of microbial fermentation (Turnbaugh et al., 2006). In this study, the proportions of Firmicutes and Bacteroidetes in the LP group were 42.90 and 53.85%, in the MP group were 64.08 and 31.18%, and in the OP group were 56.53 and 38.51%, respectively. The results showed that the energy metabolism functions, such as sugar and lipid metabolism, were higher in the MP and OP groups than in the LP group, which is consistent with the function prediction results. The menstrual cycle is based on the cyclical changes of the ovary under the action of ovarian hormones. The endometrium undergoes periodic necrosis, exfoliation, proliferation, and repair. During ovulation, the blood levels of P, androstenedione, E2, and PGF2α increase in the proximal fallopian tube, and the secretory cells become most active, which secrete various proteins and enzymes. Intestinal microbes can mediate nerve, endocrine, immune, metabolic and other pathways, and carry out two-way regulation in the brain. This interaction channel is called the microbe-gut-brain axis (Bauer et al., 2016). The communication at this axis happens through the conversion of sensory information into neural signals, hormone signals, and immune signals, which are transmitted back and forth between the central nervous system and the intestine (Mayer et al., 2015). Certain basic developmental characteristics and functions of the mammalian immune system seem to depend on the interaction with host microorganisms. The microbial population can exert pro-inflammatory and anti-inflammatory responses. The composition of the bacterial community may be closely related to the normal functioning of the immune system. For example, pregnancy is a special physiological period for women of childbearing age. During pregnancy, the levels of E2, P, and other hormones vary greatly. At the same time, under the influence of these hormones, the microbiota also undergoes changes related to the host’s physiology and immune adaptation. The remodeling of the maternal microbiota during pregnancy is an active response to change the state of the immune system, promote metabolism, and immune adaptation. Studies have reported that during a normal healthy pregnancy, the animal body undergoes a series of hormonal, immunological, and metabolic changes. These changes are accompanied by changes in the microbiota, especially in the vagina and intestines (Chen et al., 2017). A study of breastfeeding rhesus monkeys vaginal microbes during menstruation found that hormonal status affected the changes in microbiota (Hallmaier-Wacker et al., 2019). It is generally believed that autoimmune diseases are the result of complex interactions between genetic and environmental factors. Recent research reported significant differences in the microbiota of patients with autoimmune diseases, such as type I and II diabetes, systemic lupus erythematosus, Crohn’s disease, psoriasis, Behcet’s disease, and Behcet’s syndrome (Gao et al., 2008; Larsen et al., 2010; Giongo et al., 2011; Morgan et al., 2012; Edwards and Costenbader, 2014; Consolandi et al., 2015). Changes in the human microbiota, hormone levels, and immunology have a significant impact on the course of autoimmune diseases. A variety of hormones such as estrogen, prolactin, and growth hormone can regulate the activity of immune cells such as T, B, and macrophages. Regulatory B cells affect the differentiation of Th cells, promote their development into memory T cells, and inhibit pro-inflammatory T cells proliferation, thereby greatly affect the overall immune system. Th cells are classified according to cytokine expression and immune function. Th1 cells produce high levels of IL-2, IL-3, IFN-α, IFN-*γ*, TNF-β, and GMCSF. Th2 cells mediate the immune regulation of extracellular pathogens via IL-4, IL-6, IL-10, and TNF-α. For example, pregnant women with multiple sclerosis (MS) usually have a lower disease recurrence rate in the later stages of pregnancy, and the disease often becomes more severe after the delivery (Confavreux et al., 1998). Likewise, in the case of rheumatoid arthritis (RA), two-thirds of pregnant women experience improvement in RA disease (Hazes et al., 2011). Several studies indicated that intestinal microbes are related to the occurrence of neurological diseases. Intestinal microbes and their metabolites play an important role in regulating Th17/Treg balance, maintaining immune tolerance and immune response (Smith et al., 2013), For example, *Bacteroides fragilis* induces CD4 + T cells transformation into Treg cells through capsular polysaccharides and down-regulates Th17 and other pro-inflammatory factors to enhance the host immune tolerance. *Lactobacillus casei* significantly reduces the serum levels of IL-1, IL-6, IL-17, and TNF-α, while increasing the level of anti-inflammatory factors such as IL-10. This shows that microorganisms may play an important role in regulating immune system activation and tolerance. Notably, the fecal microbes of depression, PD, and AD patients all showed an increase in Bacteroidetes, while a decrease in Firmicutes; patients with sclerosis had lower enrichment of rumen bacteria (Tremlett et al., 2016). In this study, Bacteroidetes increased during the LP compared with the MP and OP, while Firmicutes and Ruminococcaceae increased during the MP and OP.

Progesterone and E2 are ovarian steroid hormones whose secretion is regulated by the hypothalamic-pituitary-ovarian axis (HPOA). Steroid hormones can also regulate the secretion of GnRH and the pituitary gland through a feedback mechanism to maintain the normal sexual cycle and reproductive function in female animals. A study on the changes in serum E2 and P levels during the menstrual cycle of cynomolgus monkeys showed that the peak of E2 appears in the FP (highest) and LP, while P hormone peaks in the LP (Ma et al., 2008). Another study showed that E2 in the urine of rhesus monkeys gradually increased in the OP, and then decreased rapidly, while P gradually increased to the peak levels in the LP (Knip and Siljander, 2016). This study only detected the hormone levels at three-time points during the menstruation cycle (MP, OP, and LP) and found that E2 reached the highest levels during the MP and the lowest in the LP; P hormone levels were the highest in the LP. These changes in E2 and P are consistent with previous reports.

This study analyzed the microbial diversity, and the function of stool samples at different stages of the menstrual cycle. It was found that the microbial diversity and function of feces changed according to the different stages of menstruation. It may be related to changes in hormone levels in the menstrual cycle, and therefore further research is needed.

## Data availability statement

The data presented in this study are deposited in the NBCI SRA (http://www.ncbi.nlm.nin.gov/sra) repository, accession number: BioProject PRJNA869047.

## Ethics statement

This animal study was reviewed and approved by Institutional Animal Care and Use Committee, IACUC, Institute of Medical Biology, Chinese Academy of Medical Sciences and Peking Union Medical College, Kunming, China.

## Author contributions

YaL and YZ conceived and designed the study, wrote the manuscript, and analyzed the sequencing data and experimental results. FY and LC performed the experiments. SD, WJ, QL, HX, WZ, YoL, and JW commented on and discussed the manuscript. ZH and YZ obtained project funding and designed, revised, and finalized the manuscript. All authors contributed to the article and approved the submitted version.
