# Supplementary material for: Intestinal microbial diversity in female rhesus (Macaca mulatta) at different physiological periods
Source: Front Microbiol. 2022 Sep 26;13:959315. doi: 10.3389/fmicb.2022.959315 (PMC9548999; doi:10.3389/fmicb.2022.959315)
Supplement: Supplementary file 1 [file Data_Sheet_1.doc]

Table 2. Animal grouping and numbering

| Group | Number | Group | Number | Group | Number |
| --- | --- | --- | --- | --- | --- |
| MP | MP-1 | OP | OP-1 | LP | LP-1 |
|  | MP-2 |  | OP-2 |  | LP-2 |
|  | MP-3 |  | OP-3 |  | LP-3 |
|  | MP-4 |  | OP-4 |  | LP-4 |
|  | MP-5 |  | OP-5 |  | LP-5 |
|  | MP-6 |  | OP-6 |  | LP-6 |

Table 3. Serum levels of E2 and P hormones in various stages of the menstrual cycle in female rhesus monkey

| Item | n | MP | OP | LP |
| --- | --- | --- | --- | --- |
| E2(pg/mL) | 6 | 60.79±18.66^a^ | 44.66±26.40^ab^ | 37.82±15.75^b^ |
| P(ng/mL) | 6 | 0.14±0.03^a^ | 1.82±1.39^b^ | 4.17±2.46^b^ |

Note: Three groups of data comparison, for the same detection index, marked with the same letter means no significant difference (P > 0.05), different letters means significant difference (P < 0.05).


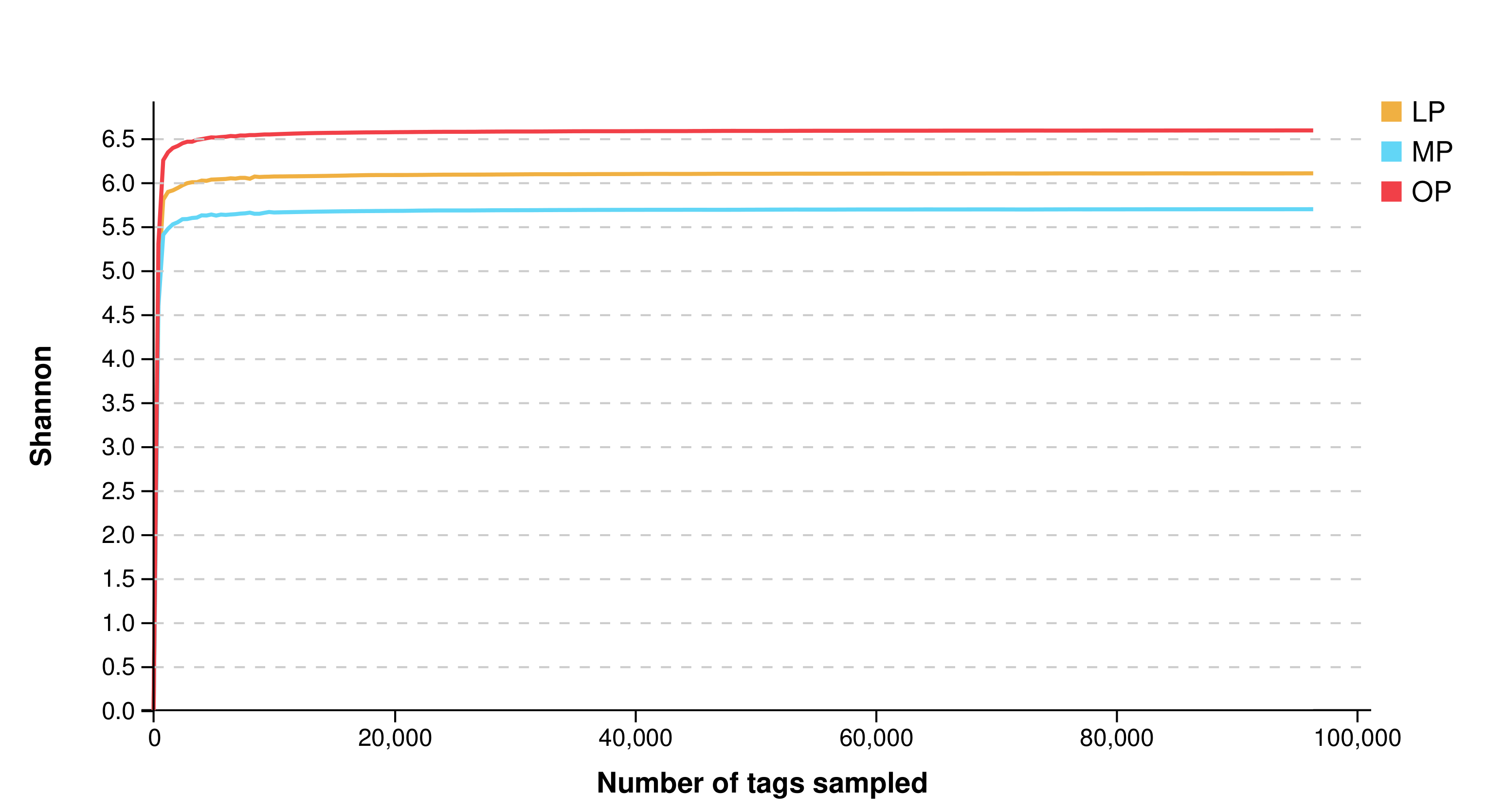


Fig 6. Shannon exponential dilution curve.


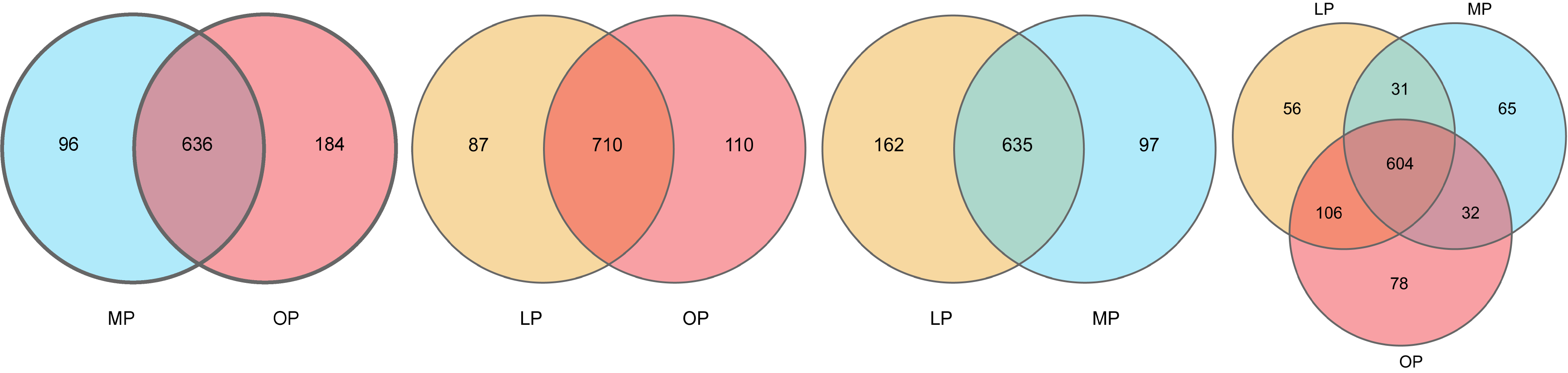


Fig 7. The number of OUTs in feces samples at the respective stages of the menstrual cycle.


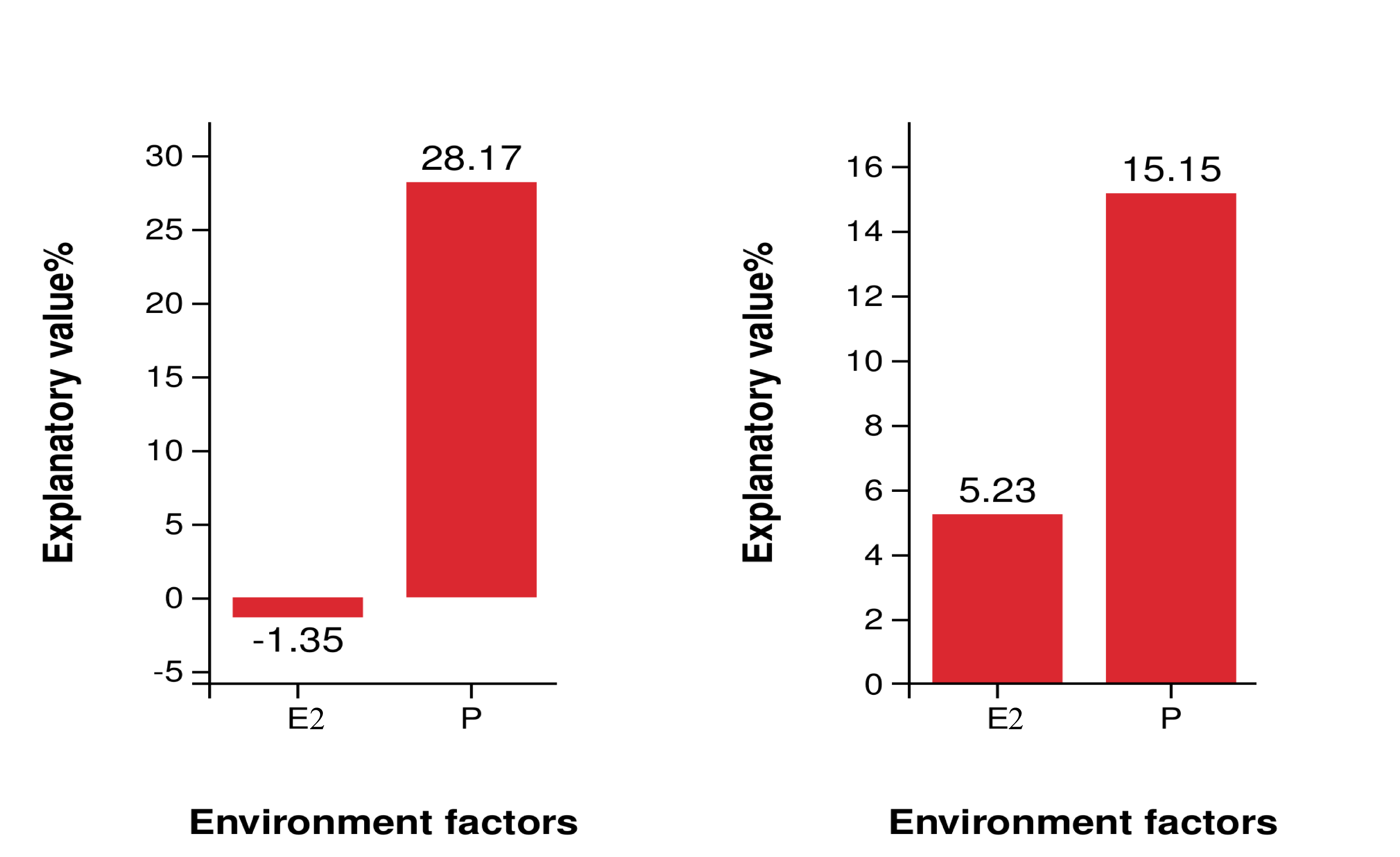


Fig 8. Environmental contribution at the phylum and genus levels.


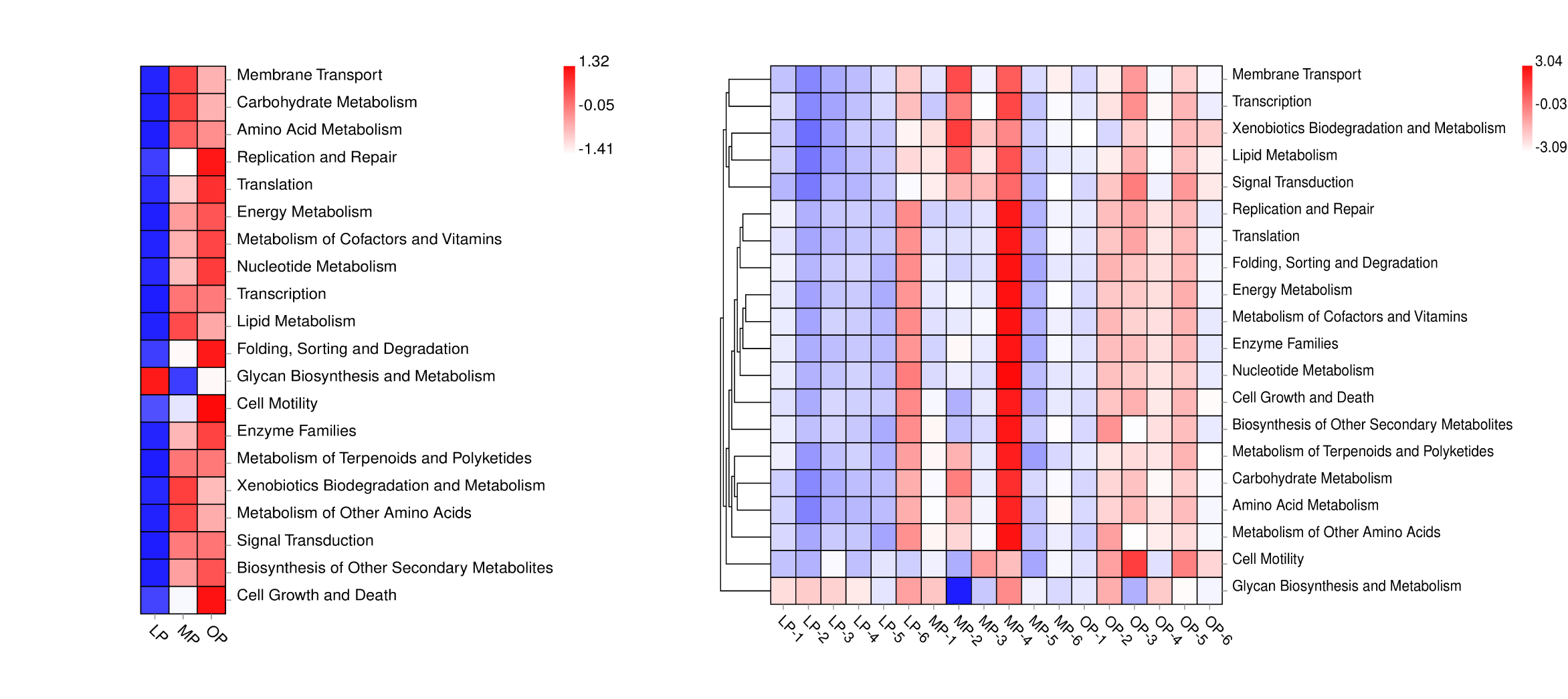


Fig 9.Heat map of relative abundance of different functions at different stages of the menstrual cycle and between samples at different stages of the menstrual cycle.
